# Relationship between weight of rescuer and quality of chest compression during cardiopulmonary resuscitation

**DOI:** 10.1186/1880-6805-33-16

**Published:** 2014-06-24

**Authors:** Tomoyuki Hasegawa, Rie Daikoku, Shin Saito, Yayoi Saito

**Affiliations:** 1 Graduate School of Health Care Sciences, Tokyo Medical and Dental University, 1-5-45 Yushima Bunkyo-ku, Tokyo 113-8510, Japan; 2 Mie Prefectural College of Nursing, 1-1-1 Yumegaoka, Tsu-shi, Mie 514-0116, Japan

**Keywords:** Cardiopulmonary resuscitation, Chest compression, Rescuer’s physique, Fatigue, Rotation time, Weight, Nurse

## Abstract

**Background:**

According to the guidelines for cardiopulmonary resuscitation (CPR), the rotation time for chest compression should be about 2 min. The quality of chest compressions is related to the physical fitness of the rescuer, but this was not considered when determining rotation time. The present study aimed to clarify associations between body weight and the quality of chest compression and physical fatigue during CPR performed by 18 registered nurses (10 male and 8 female) assigned to light and heavy groups according to the average weight for each sex in Japan.

**Methods:**

Five-minute chest compressions were then performed on a manikin that was placed on the floor. Measurement parameters were compression depth, heart rate, oxygen uptake, integrated electromyography signals, and rating of perceived exertion. Compression depth was evaluated according to the ratio (%) of adequate compressions (at least 5 cm deep).

**Results:**

The ratio of adequate compressions decreased significantly over time in the light group. Values for heart rate, oxygen uptake, muscle activity defined as integrated electromyography signals, and rating of perceived exertion were significantly higher for the light group than for the heavy group.

**Conclusion:**

Chest compression caused increased fatigue among the light group, which consequently resulted in a gradual fall in the quality of chest compression. These results suggested that individuals with a lower body weight should rotate at 1-min intervals to maintain high quality CPR and thus improve the survival rates and neurological outcomes of victims of cardiac arrest.

## Background

Cardiopulmonary resuscitation (CPR) comprises a series of lifesaving actions that has traditionally integrated chest compression and breathing to optimize circulation and oxygenation to improve the likelihood of survival after cardiac arrest [1,2]. The 2010 American Heart Association (AHA) guidelines for CPR and emergency cardiovascular care (ECC) suggest that all rescuers, regardless of training, should provide chest compression to victims of cardiac arrest [1]. Providing early effective chest compression for such victims improves the chances of both survival and neurologically favorable outcomes [3-6].

Uninterrupted chest compression causes physical fatigue in rescuers and decreases the number of adequately deep chest compressions [7,8]. Current guidelines emphasize the importance of pushing hard and fast and of minimizing interruptions during compression [1]. Therefore, following these guidelines will result in a rapid decline in the quality of chest compression. If more rescuers are available, they should rotate the application of compression every 2 min. Although the quality of chest compressions is related to the physical fitness of the rescuer [9-11], the guidelines do not consider this when determining the rotation time.

Most cardiac arrests in hospitals are witnessed by nurses [12], whose key role in such emergencies is to apply initial CPR. Most institutions require that nurses be trained in basic life support, since nursing competence in CPR critically affects the outcomes of cardiac arrest [13]. The total number of nurses practicing in Japan during 2011 was 1,027,337, about 95% of whom are women [14] and about half of them are aged in their 20s or 30s. Any individual in a hospital should be able to provide the best possible quality chest compression regardless of physical type. Therefore, rotation time rather than uniform rotation time considering the physique of rescuer should be considered to ensure the most effective chest compression. The quality of chest compression positively correlates with the height of the rescuer [10], but the relationship with the weight of the rescuer has not been proven. The Japanese physique is generally smaller than that of Europeans and Americans and although the weight of rescuers might influence the quality of chest compression, rotation time should be determined according to a smaller physique.

The present study aimed to clarify associations between the quality of chest compression and body weight as well as the physical fatigue of rescuers. We propose that the amount of rotation time required to deliver the most effective chest compression delivered by persons with a lower body weight should be decreased to deliver more effective cardiopulmonary resuscitation to ensure optimal outcomes for victims of cardiac arrest.

## Methods

### Participants

The present study included 18 (male, n = 10; female, n = 8) registered nurses employed in emergency, intensive care, or cardiovascular medicine departments who have current certifications in basic life support and have delivered in-hospital CPR. None of them had musculoskeletal or functional mobility issues. All of them provided written, informed consent to participate in this study, which was approved by the Mie Prefectural College of Nursing. Information was then collected about sex, age, clinical experience, height, weight, body mass index (BMI), and exercise habits (Table 1). None of the nurses regularly participated in exercise.

**Table 1:** Characteristics of participants

	**Male**	**Female**	**Total**
	**n = 10**	**n = 8**	**n = 18**
Age (years)	28.1 ± 3.6	29.1 ± 2.3	28.5 ± 3.1
Height (cm)	173.8 ± 6.3	158.0 ± 4.2	166.8 ± 9.6
Weight (kg)	64.5 ± 10.6	53.4 ± 10.2	59.7 ± 11.8
BMI (kg/m^2^)	21.3 ± 3.8	21.4 ± 3.9	21.3 ± 3.8

Values are means ± standard deviation.

### Protocol

**Figure 1:**
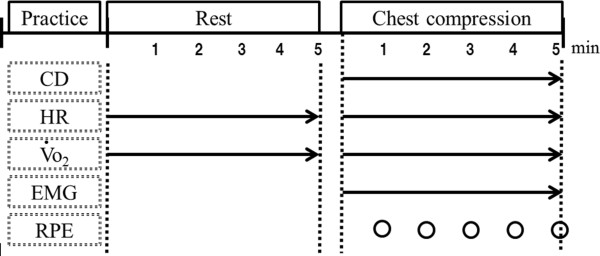
**Experimental protocol.** Compression depth (CD), heart rate (HR), oxygen uptake (VO2), electromyography (EMG), and rating of perceived exertion (RPE) were measured.

**Figure 2:**
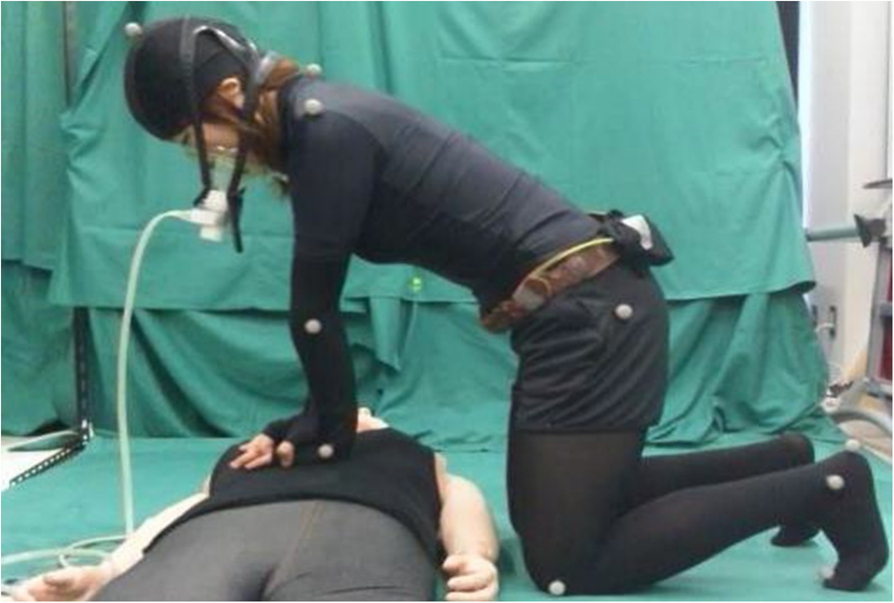
Posture for chest compression.

This study protocol comprised rest and chest compression in that order for 5 min each (Figure 1). The nurses practiced chest compression until they could consistently apply at least 5 cm of compression. During the 5-min rest period, they remained seated with their eyes closed while wearing a face-mask and electromyography (EMG) and electrocardiography (ECG) electrodes. Five-minute chest compressions were then performed on a Resusci Anne Skill Reporter (Leardal Medical Corporation, Stavanger, Norway) that was placed on the floor without audiovisual feedback (Figure 2). They maintained a compression rate of 100/min by following a metronome [15]. At the end of the chest compressions, they recovered in a comfortable posture.

### Data collection

Compression depth (CD) was captured as each participant performed CPR on a Resusci Anne Skill Reporter. Heart rate was measured at rest and during chest compression using a Life Scope 8 (NIHON KOHDEN Co., Tokyo, Japan). Oxygen uptake (VO2) during chest compression was continuously measured using a VO2000 (S&ME Co., Raleigh, NC, USA). Surface electromyography (sEMG) captured data from the biceps brachii, triceps brachii, trapezius, erector spinae, external oblique muscle, abdominal rectus muscle, rectus femoris, and biceps femoris during chest compression [16,17]. Two electrodes were attached to the belly of each muscle at an inter-electrode distance of 2.5 cm. The skin was abraded and cleaned with alcohol before attaching electrodes to minimize impedance. Analog HR and EMG signals were sampled using an AD16-16(PCI) E A/D converter (CONTEC CO. Ltd., Osaka, Japan) at 3 KHz, and stored in a personal computer using a G1 system Analog Recorder Pro Ver. 1.60 (G1 system, Aichi, Japan). The rating of perceived exertion (RPE) was rated using Borg’s 15 point scale (range, 6 to 20).

### Evaluation

The nurses were assigned to light (5 male and 4 female) and heavy (male, n = 5; female, n = 4) groups according to the average weight for each sex in Japan [18]. Compression depth (CD) was evaluated according to the ratio (%) of adequate compressions (at least 5 cm deep) per 30 s [19]. Heart rate and VO2 were evaluated during both chest compression based on changes from the initial baseline value after rest. The sEMG signals were full-wave rectified and integrated over a period of 30 s during chest compressions to determine muscle activity (iEMG) [17]. Physical fatigue was determined by matching against an RPE scale during chest compression every minute.

### Statistical analysis

The iEMG values were compared between the heavy and light groups using independent-sample t tests and a one-way analysis of variance. The ratio (%) of adequate compressions as well as values for HR, VO2 and RPE were analyzed using the Mann-Whitney U test, the Friedman test, and the Wilcoxon sign rank test. All data were statistically analyzed using SPSS/PASW Statistics Ver. 18.0 (IBM, Armonk, NY, USA). The significance level was set at 0.05.

### Ethics

The Ethics Review board at Mie Prefectural College of Nursing approved the study (Approval No. 120203). The experimental protocols and procedures were explained to all those who responded to public advertisements about the study and then all voluntarily provided written, informed consent to participate. High priority was given to the safety of the participants and appropriate measures were taken to process the withdrawal of any participant from the study at any time.

## Results

### Participants

The average weight and BMI of the light and heavy groups significantly differed (50.6 ± 6.5 *vs.* 68.0 ± 7.5 kg, 18.2 ± 1.6 *vs.* 24.4 ± 2.8 kg/m^2^, *P* <0.001; Table 2), whereas age, clinical experience, height, HR, and VO2 at rest did not. All participants were capable of applying chest compression for 5 min.

**Table 2:** Characteristics of groups

	**Light**			**Heavy**			
	**Male**	**Female**	**Total**	**Male**	**Female**	**Total**	***P***
	**n = 5**	**n = 4**	**n = 9**	**n = 5**	**n = 4**	**n = 9**	
Age (years)	28.2 ± 2.1	30.2 ± 2.4	30.0 ± 2.9	26.4 ± 3.0	28.0 ± 1.6	27.1 ± 2.6	ns
CE (years)	7.3 ± 4.3	6.8 ± 2.7	7.0 ± 3.9	6.3 ± 4.7	5.7 ± 1.7	6.0 ± 3.9	ns
Height (cm)	172.3 ± 5.5	158.7 ± 2.8	166.2 ± 8.1	175.3 ± 6.6	157.3 ± 5.2	167.3 ± 10.8	ns
Weight (kg)	55.3 ± 4.8	44.7 ± 1.7	50.6 ± 6.5	73.0 ± 4.9	61.9 ± 5.5	68.0 ± 7.5	***
BMI (kg/m^2^)	18.9 ± 2.0	17.7 ± 0.5	18.2 ± 1.6	23.9 ± 3.2	25.0 ± 2.0	24.4 ± 2.8	***
HR (bpm)	78.1 ± 5.1	70.2 ± 5.4	74.1 ± 7.0	69.0 ± 10.1	77.0 ± 5.8	73.0 ± 10.1	ns
VO2 (mL/kg/min)	4.0 ± 1.0	2.8 ± 0.7	3.4 ± 1.2	3.0 ± 0.3	2.6 ± 0.4	2.8 ± 0.4	ns

Values are means ± standard deviation.

***P <0.001 (unpaired *t* test) between totals of light and heavy groups.

CE, clinical experience; ns, no significance.

### Compression depth

**Figure 3:**
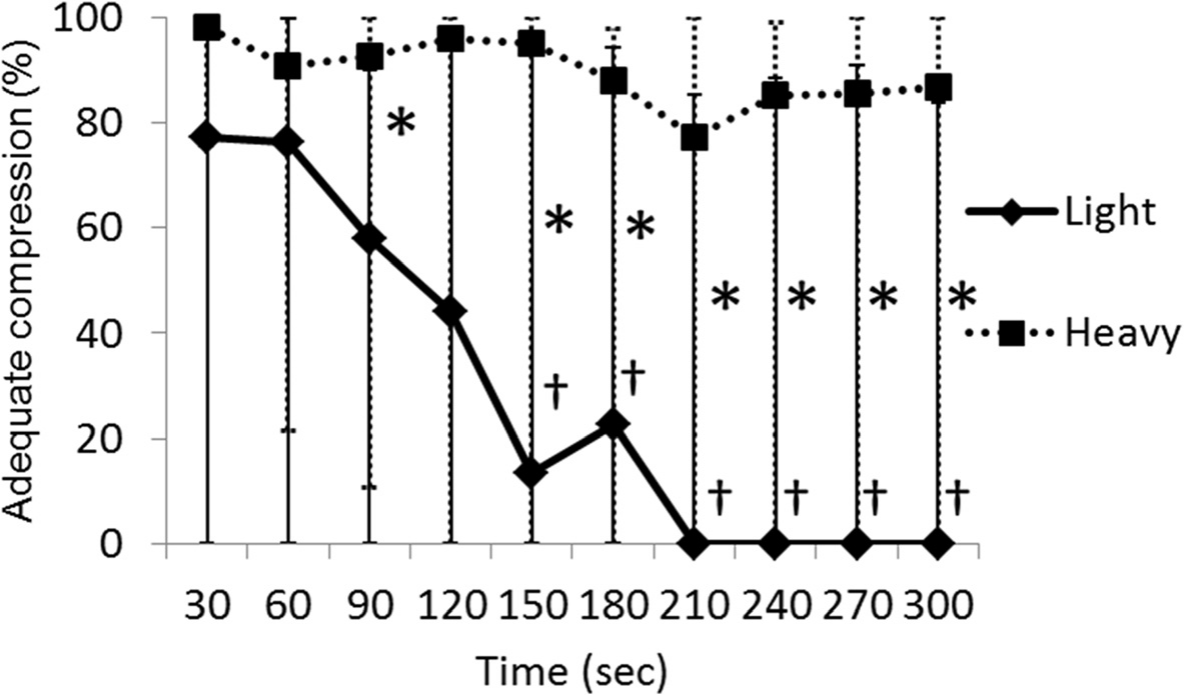
**Comparison of ratios of adequate compression between light and heavy groups.** Values are shown as medians and max-min. Significant differences between light and heavy groups (*P <0.05; Mann-Whitney U test) and between 30 s and subsequent times in light group (^†^*P* <0.05; Wilcoxon sign rank test).

The median ratios (%) of adequate compression for each 30-s interval by the light and heavy groups ranged from 77.4 to 0.0 and 98.1 to 77.3, respectively (Figure 3). The ratio of adequate compression applied by the heavy group did not significantly decline, but significantly decreased over time from 150, 180, 210, 240, 270, and 300 s (*P* = 0.028) in the light group. Values significantly differed at 90, 150, 180, 210, 240, 270, and 300 s (*P* = 0.027, 0.049, 0.049, 0.035, 0.035, 0.022, 0.035, respectively) between the groups.

### Heart rate

**Figure 4:**
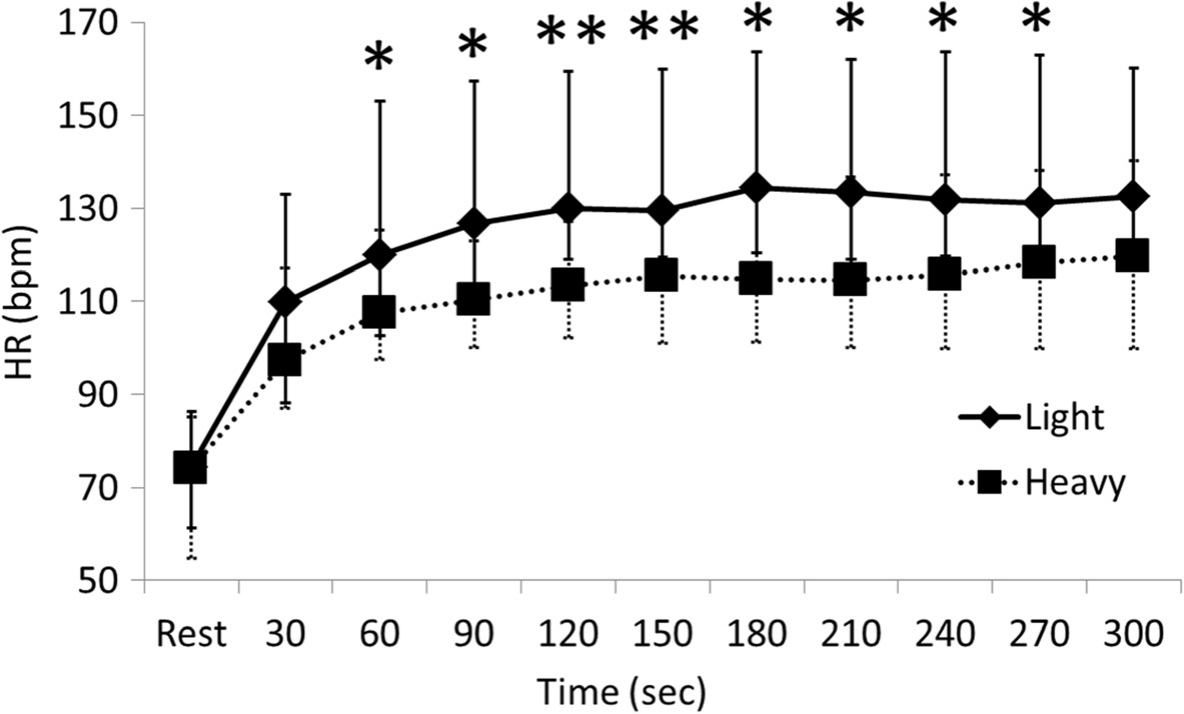
**Comparison of heart rates between light and heavy groups.** Values are shown as medians and max-min. Significant difference between light and heavy groups (*P <0.05 and **P <0.01; Mann-Whitney U test). HR, heart rate.

The median value of HR (bpm) for chest compression compared with that at rest for each 30-s interval ranged from 134.3 to 109.9 and 118.2 to 97.2 in the light and heavy groups, respectively (Figure 4). These values significantly differed between the groups at 60, 90, 120, 150, 180, 210, 240, and 270 s (*P* = 0.043, 0.020, 0.008, 0.008, 0.013, 0.029, 0.029, 0.043, respectively) during chest compression.

### Oxygen uptake

**Figure 5:**
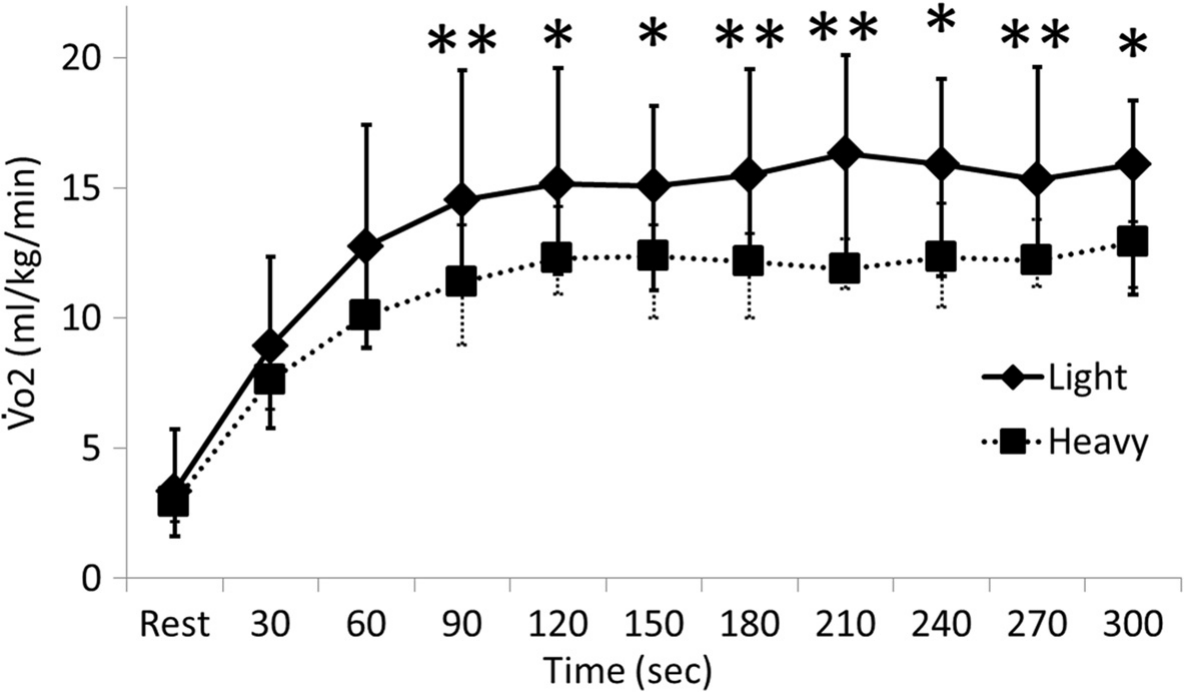
**Comparison of oxygen uptake between light and heavy groups.** Values are shown as medians and max-min. Significant difference between light and heavy groups (*P <0.05 and **P <0.01; Mann-Whitney U test). VO2, oxygen uptake.

The median value of VO2 (mL/kg/min) for chest compression compared with that at rest for each 30-s interval ranged from 16.3 to 8.9 and 12.9 to 7.6 in the light and heavy groups, respectively (Figure 5). Values significantly differed between the groups during chest compression at 90, 120, 150, 180, 210, 240, 270, and 300 s (*P* = 0.005, 0.020, 0.014, 0.007, 0.007, 0.014, 0.005, 0.020, respectively).

### Muscle activity

Mean iEMG (mV) values for the trapezius, erector spinae, external oblique muscle, abdominal rectus muscle, and rectus femoris significantly differed between the light and heavy groups (Table 3), whereas those for the biceps brachii, triceps brachii, and biceps femoris did not significantly differ between the groups. Muscle activity did not significantly change over time against the value after 30 s in either group.

**Table 3:** Comparison of iEMG values between light and heavy groups

	**30**	**60**	**90**	**120**	**150**	**180**	**210**	**240**	**270**	**300 (s)**
Biceps brachii (mV)										
Light	27.3 ± 12.8	26.5 ± 12.5	24.5 ± 10.1	25.1 ± 11.0	23.5 ± 9.5	24.3 ± 9.6	22.2 ± 8.7	23.9 ± 10.1	22.2 ± 9.7	23.2 ± 10.6
Heavy	28.5 ± 9.5	26.2 ± 6.9	24.0 ± 6.2	23.2 ± 6.9	22.7 ± 6.9	22.7 ± 7.4	22.2 ± 8.0	22.9 ± 8.7	22.9 ± 9.1	22.3 ± 9.2
Triceps brachii (mV)										
Light	69.5 ± 25.6	67.4 ± 22.5	65.7 ± 20.5	65.6 ± 18.2	67.3 ± 18.8	70.3 ± 18.8	68.7 ± 18.2	68.6 ± 17.0	68.4 ± 15.4	70.4 ± 16.8
Heavy	50.1 ± 9.0	51.1 ± 10.3	50.4 ± 11.3	52.0 ± 12.7	51.2 ± 13.5	52.5 ± 16.0	53.0 ± 16.0	55.2 ± 18.9	53.7 ± 17.0	55.8 ± 16.5
Trapezius (mV)										
Light	26.8 ± 14.8	25.1 ± 14.5	21.9 ± 11.2	21.0 ± 10.2	20.8 ± 9.6	21.9 ± 9.4	21.1 ± 8.0	22.3 ± 9.2	21.8 ± 7.2	25.5 ± 10.1
Heavy	14.3 ± 4.8*	13.7 ± 4.7	13.3 ± 4.9	13.5 ± 5.4	14.2 ± 9.0	13.6 ± 7.9	12.7 ± 5.2*	12.1 ± 3.9*	12.3 ± 5.4**	13.3 ± 5.8**
Erector spinae (mV)										
Light	19.0 ± 5.4	19.8 ± 6.3	19.4 ± 5.7	19.1 ± 5.0	18.7 ± 5.1	19.5 ± 5.4	19.1 ± 5.2	20.0 ± 4.8	20.0 ± 4.7	20.5 ± 4.4
Heavy	12.2 ± 4.9*	12.8 ± 6.0*	13.0 ± 5.4*	12.7 ± 4.8*	12.9 ± 4.5*	12.9 ± 3.9*	13.1 ± 4.3*	13.2 ± 4.3**	13.2 ± 4.8**	13.4 ± 5.1**
External oblique (mV)										
Light	22.0 ± 9.4	25.9 ± 14.2	22.6 ± 12.0	22.4 ± 11.8	19.6 ± 8.8	21.6 ± 9.6	20.6 ± 8.8	22.2 ± 10.2	21.0 ± 9.2	22.0 ± 10.5
Heavy	11.3 ± 6.3*	10.9 ± 5.4*	10.3 ± 4.9*	9.6 ± 4.1*	9.5 ± 4.6*	9.5 ± 4.4**	9.3 ± 4.5**	9.1 ± 4.1**	8.6 ± 3.9**	8.3 ± 3.2**
Abdominal rectus (mV)										
Light	18.4 ± 6.7	19.3 ± 10.3	18.2 ± 9.6	18.4 ± 11.2	17.3 ± 10.0	18.1 ± 9.5	17.3 ± 9.5	17.9 ± 10.7	17.6 ± 9.3	17.7 ± 9.5
Heavy	8.7 ± 3.5**	8.4 ± 3.3*	8.0 ± 2.7*	7.7 ± 2.7*	7.0 ± 2.4*	7.2 ± 2.4**	7.0 ± 2.5**	7.2 ± 2.8*	7.0 ± 2.6**	7.3 ± 2.9*
Rectus femoris (mV)										
Light	7.2 ± 2.3	8.6 ± 4.1	10.6 ± 5.6	11.1 ± 6.7	10.5 ± 6.8	11.2 ± 7.2	9.7 ± 5.9	10.2 ± 7.1	10.7 ± 6.5	11.1 ± 7.9
Heavy	4.3 ± 0.8**	4.1 ± 0.7*	3.7 ± 0.7**	3.7 ± 0.7*	3.5 ± 0.7*	3.7 ± 1.1*	3.4 ± 0.9*	3.7 ± 1.1*	3.9 ± 1.7*	3.5 ± 0.8*
Biceps femoris (mV)										
Light	32.3 ± 24.8	28.5 ± 18.2	24.0 ± 17.1	22.1 ± 15.3	23.3 ± 15.4	23.8 ± 15.5	24.3 ± 16.7	26.4 ± 16.7	26.4 ± 17.1	26.8 ± 15.7
Heavy	14.1 ± 6.6	15.2 ± 7.1	15.1 ± 5.6	15.1 ± 5.5	15.3 ± 5.5	14.7 ± 5.4	14.5 ± 4.8	14.5 ± 4.9	14.6 ± 4.5	14.5 ± 4.2

Values are means ± standard deviation. Significant difference between light and heavy groups. (*P <0.05, **P <0.01; unpaired *t* test); n = 9 for all groups.

### Rating of perceived exertion

**Figure 6:**
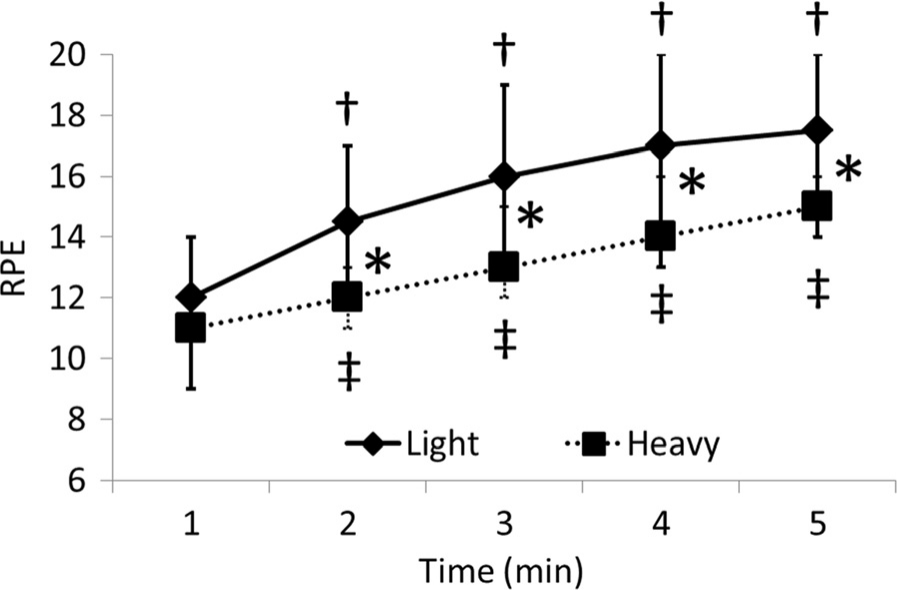
**Comparison of RPE between light and heavy groups.** Values are shown as medians and max-min. Significant difference between light and heavy groups (*P <0.05; Mann-Whitney U test) and between 1 min and subsequent times in light (^†^*P* <0.05; Wilcoxon sign rank test) and heavy (^‡^*P* <0.05; Wilcoxon sign rank test) groups. RPE, rating of perceived exertion.

The median values for rating of perceived exertion (RPE) for each 1-min interval ranged from 17.5 to 12.0 and 15.0 to 11.0 in the light and heavy groups, respectively (Figure 6). The RPE significantly increased over time against the value for the first minute for 2, 3, 4, and 5 in both groups. Values significantly differed between the groups at 2, 3, 4, and 5 min (*P* = 0.014, 0.010, 0.021, 0.018, respectively).

## Discussion

The quality of chest compression delivered during CPR using only the hands decreases over time [1-3,8,16,19,20]. The present study confirmed that the quality of chest compression by the light group changed as described [7,8] and this group became more fatigued thereafter.

Chest compression requires power to be applied from a point that is vertically above the sternum to a depth of 5 cm. The amount of power required to depress a sternum by 5 cm is about 500 N [21-23]. Chest compression force during CPR is generated using gravity and hip flexion torque [16]. Those applying chest compression develop force by accelerating the upper body downwards using gravity [16] and use hip extension torque to hold the trunk up at decompression, which resists the inertial force of gravity [16]. The moment acting at the lumbar spine represents the loads generated by muscles during chest compression [24].

The light group produced the required force for pressure by utilizing the trapezius, abdominal rectus, external oblique, and rectus femoris muscles. At the moment of decompression, force enters the erector spinae, which is an antagonist of compression, and this might explain the increased physical fatigue in the light group compared with the heavy group.

For this reason, a lighter rescuer quickly becomes subjectively and objectively fatigued in during hands-only CPR, and the quality of chest compression rapidly deteriorates. Furthermore, one of our participants was unable to compress the sternum to a depth of 5 cm from the start of the experiment. Either debriefing or feedback alone improves CPR quality, but the combination of both improves performance more effectively [25]. An audiovisual cardiopulmonary resuscitation feedback device has significantly improved the quality of chest compression provided by experienced hospital nurses in a simulated setting [26]. We did not use an audiovisual feedback device in the present study and assumed that some participants in the light group had forgotten what they had learned while practicing chest compression before starting the experiments.

On the other hand, the heavy group maintained the ratio of adequate compression at >70% for 5 min. This group was able to generate sufficient compression force without having to involve the muscular power of the trunk or thigh, unlike the light group. Chest compression by the heavy group utilized the rescuer’s weight as a compression force. Good physical fitness and the height of the rescuer correlate positively and independently of sex with the quality of chest compression [10,11]. Our data are in agreement with these findings. The heavy group could use their body weight to provide chest compression without becoming quickly fatigued, indicating that the weight of the rescuer is an important element of effective chest compression.

Effective chest compressions are essential for providing blood flow during CPR. For this reason all patients in cardiac arrest should receive chest compression [1]. To provide effective chest compression, push hard and push fast. It is reasonable for laypersons and healthcare providers to compress the adult chest at a rate of at least 100 compressions per minute with a compression depth at least 5 cm [1]. Chest compression in hospital is often insufficient even when applied by medical staff [27]. According to the guidelines, the rotation time for chest compression should be about 2 min, regardless of physical type. However, the physical features of rescuers must be considered to provide and maintain high quality CPR. With respect to the rotation time for chest compression in particular, an index could be constructed using the body weight of rescuers as one factor. This study suggests that rescuers of low weight should rotate every minute to maintain effective CPR.

## Conclusions

The light group became fatigued while delivering chest compression, which gradually decreased the quality of the compression. On the other hand, the heavy group could apply effective chest compression for 5 min. The weight of the rescuer is an important factor in the quality of chest compression. The ratio of adequate compression significantly differed after 1 min, suggesting that individuals of light weight should rotate at intervals of 1 min.

## Abbreviations

AHA: American Heart Association; BMI: Body mass index; CD: Compression depth; CE: Clinical experience; CPR: Cardiopulmonary resuscitation; ECC: Emergency cardiovascular care; ECG: Electrocardiogram; EMG: Electromyogram; HR: Heart rate; iECG: Integrated electrocardiograph; RPE: Rating of perceived exertion; sECG: Surface electrocardiography; VO2: oxygen uptake.

## Competing interests

The authors declare that they have no competing interests.

## Authors’ contributions

TH performed the experiments, analyzed the data and wrote the manuscript. DR, SS, and YS coordinated the study and supervised the collection and analysis of data. All authors have read and approved the final manuscript.
